# Staff-Reported Peri-Procedural Workflow Vulnerabilities and a Preliminary Checklist Prototype for Mechanically Ventilated Intensive Care Patients Undergoing Hyperbaric Oxygen Therapy: A Single-Centre Pilot Survey

**DOI:** 10.3390/jcm15145418

**Published:** 2026-07-10

**Authors:** Aneta Miszewska, Jacek Kot

**Affiliations:** 1 Division of Anaesthesiology Nursing & Intensive Care, Medical University of Gdańsk, ul. Dębinki 7, Building 15, 80-211 Gdansk, Poland; 2 Department of Hyperbaric Medicine and Sea Rescue, University Centre for Maritime and Tropical Medicine, ul. Powstania Styczniowego 9b, 81-519 Gdynia, Poland; jkot@gumed.edu.pl; 3 National Centre for Hyperbaric Medicine, Institute of Maritime and Tropical Medicine, Medical University of Gdańsk, ul. Powstania Styczniowego 9b, 81-519 Gdynia, Poland

**Keywords:** hyperbaric oxygen therapy, critically ill patient, peri-procedural management, patient safety, checklist, intensive care nursing

## Abstract

**Background**: Peri-procedural management of mechanically ventilated intensive care unit (ICU) patients undergoing hyperbaric oxygen therapy (HBOT) requires coordinated preparation, chamber-compatible equipment management, transfer readiness, and restoration of standard ICU support. Evidence on HBOT-specific workflow vulnerabilities remains limited. This single-centre pilot survey described staff-reported observations of omitted, delayed, or incompletely performed peri-HBOT tasks and used them to inform a preliminary checklist prototype. **Methods**: Nineteen healthcare professionals involved in peri-HBOT care at a specialist hyperbaric centre in Poland completed an anonymous questionnaire based on one-month retrospective recall. Respondents indicated whether they had observed each listed task being omitted or incompletely performed at least once. Analyses used respondent-level counts, percentages, and Wilson 95% confidence intervals. **Results**: Before HBOT, the most frequently reported observations concerned preparation of the intubation set for transfer (14/19, 73.7%; 95% CI: 51.2–88.2), disconnection of the anti-decubitus mattress pump (8/19, 42.1%; 95% CI: 23.1–63.7), removal or replacement of hazardous bed materials (7/19, 36.8%; 95% CI: 19.1–59.0), and preparation of the self-inflating bag (6/19, 31.6%; 95% CI: 15.4–54.0). After HBOT, observations most often involved replacing fluid with air in the endotracheal tube cuff (11/19, 57.9%; 95% CI: 36.3–76.9), reconnecting the anti-decubitus mattress pump (10/19, 52.6%; 95% CI: 31.7–72.7), and reconnecting interrupted intravenous infusions (5/19, 26.3%; 95% CI: 11.8–48.8). **Conclusions**: Findings identified perceived peri-HBOT workflow vulnerabilities and informed a locally derived checklist prototype. They should not be interpreted as verified omission prevalence, event rates, patient harm, or checklist effectiveness; usability testing, refinement, and multicentre validation are required.

## 1. Background

Hyperbaric oxygen therapy (HBOT) is a form of treatment involving breathing 100% oxygen for at least 60 min, during which the partial pressure of oxygen exceeds 1.5 absolute atmospheres (ATA) [1]. HBOT is also used in critically ill patients in conditions such as carbon monoxide (CO) poisoning, necrotizing soft tissue infections (NSTI), crush injuries, air or gas embolism, severe neurological decompression illness, and acute thermal burns [2].

The hyperbaric environment introduces multiple factors that may increase the risk of adverse events [3]; therefore, it is essential that the care of critically ill patients undergoing HBOT be provided by appropriately trained personnel [1,4]. Intrahospital transport of critically ill patients is an established patient-safety domain, and cardiovascular and respiratory events are among the most common complications during transfer [5]. A previous retrospective study from our centre identified risk factors for adverse events during both transfer and HBOT sessions in intensive care patients [3]. Accordingly, in mechanically ventilated ICU patients undergoing HBOT, safety should be considered across the entire peri-procedural pathway rather than during transport alone [3]. This pathway includes chamber-specific preparation, adaptation of equipment to the hyperbaric environment, the HBOT session itself, and restoration of standard ICU support after the session [6,7]. Continuous monitoring of vital signs and structured protocols are therefore essential throughout peri-HBOT management [4,6]. Moreover, it is recommended that medical personnel perform at least 100 HBOT sessions annually with critically ill patients to maintain proficiency in hyperbaric patient transport and management [4]. This process is also highly relevant to nursing practice. Nurses play a central role in airway security, monitoring, infusion management, device preparation, transport readiness, and continuity of care before, during, and after HBOT.

The HBOT environment also affects the work system around the patient. During treatment sessions, attending staff may experience reduced cognitive performance [2,8], while critical-care work is already characterised by workload, time pressure, shift work, and reliance on coordinated team performance [9]. From a human factor perspective, peri-procedural safety should therefore be viewed as a property of the work system rather than as the result of individual vigilance alone. Systems-based patient-safety models emphasise interactions between clinicians, tasks, equipment, technologies, the physical environment, organisational routines, and feedback mechanisms [10]. In this context, omitted, delayed, or incompletely performed tasks may be interpreted as potential workflow vulnerabilities rather than isolated individual failures.

Checklists may support complex ICU work by acting as cognitive and communication aids that promote completion of essential task components, support adherence to best practice, and reduce errors of omission [11]. In intensive care, checklists have been used for ward rounds, delirium screening, transfer and handover, infection prevention, and airway management [11]. Recent ICU review evidence suggests that checklists may improve selected patient-related and process-of-care outcomes, although certainty is limited by heterogeneity in checklist design, implementation, validation, and outcome measurement [11,12]. However, checklist value depends not on the existence of a form alone, but on usability, item relevance, avoidance of redundancy, explicit task ownership, workflow fit, leadership, feedback, stakeholder engagement, and local adaptation [11,13,14,15]. This is particularly relevant to peri-HBOT care because generic ICU transport checklists may not fully address chamber-compatible equipment, material-safety constraints, pressure-related airway-management issues, or post-session restoration of standard ICU support.

The study was conducted at the Department of Hyperbaric Medicine and Sea Rescue at the University Centre for Maritime and Tropical Medicine in Gdynia (hereinafter referred to as the Hyperbaric Centre, HC), a specialist hyperbaric centre in which critically ill patients requiring HBOT are hospitalised in the same building as the intensive care unit. Patients are accommodated on hyperbaric-adapted beds, transfers to the chamber are short and intra-departmental, and care is maintained by the same trained hyperbaric-ICU team before, during, and after HBOT. This integrated model differs from longer, remote, or handover-based HBOT transfer pathways, but it still requires deliberate preparation of the patient, airway, equipment, monitoring, chamber environment, and post-session restoration of standard ICU support [3,4].

Despite available guidance on critical-care HBOT and generic ICU transport safety, evidence specific to peri-procedural preparation and post-session stabilisation of mechanically ventilated ICU patients undergoing HBOT remains limited. To our knowledge, no internationally accepted or validated checklist exists for this pathway, and generic ICU transport checklists do not fully address HBOT-specific environmental, technical, and pressure-related constraints.

The present pilot study therefore aimed to describe staff-reported, respondent-level observations of omitted, delayed, or incompletely performed peri-procedural tasks in mechanically ventilated ICU patients undergoing HBOT, with particular attention to pre-session preparation and post-session stabilisation. A secondary aim was to use these observations, together with local workflow review and literature-informed rationale, to inform a locally derived preliminary checklist prototype.

## 2. Materials and Methods

### 2.1. Design

This was a single-centre, observational, descriptive pilot study based on a diagnostic survey conducted using an anonymous, self-administered questionnaire developed specifically for this research. Although data collection was performed during a predefined study period, the questionnaire relied on respondents’ retrospective recall of omissions observed during the preceding month.

### 2.2. Study Setting and Sampling

The study was conducted at the Department of Hyperbaric Medicine and Sea Rescue at the University Centre for Maritime and Tropical Medicine in Gdynia (Hyperbaric Centre, HC). The survey was distributed among ICU nurses and physicians trained in hyperbaric medicine and involved in peri-HBOT patient preparation, transfer, and care at the HC. In total, 19 of 23 eligible staff members (13 nurses and 6 physicians) completed the survey. The unit of analysis in this study was the individual staff respondent rather than the patient, HBOT session, or transfer episode. During the survey period, 26 HBOT sessions involving intensive care patients were conducted at the centre; this number is reported only to describe clinical activity during the study period and was not used as the denominator for analysis. The survey was anonymous and did not collect patient-level or session-level identifiers, the number of unique patients, whether multiple HBOT sessions involved the same patient, and whether more than one respondent referred to the same omission event could not be determined. Because no adverse events, patient-level outcomes, or independently verified process failures were recorded, the study was not designed to determine whether the reported omissions translated into measurable clinical harm.

### 2.3. Inclusion Criteria

Inclusion criteria were as follows: employment at the HC for at least three months (post-adaptation period), professional role as a nurse or physician, and involvement in the care of mechanically ventilated patients.

### 2.4. Survey Instrument Development and Internal Face/Content Review

The data source was an anonymous, self-administered questionnaire developed specifically for this exploratory pilot survey. The instrument was designed to capture staff-reported observations of peri-HBOT preparation and post-session stabilisation tasks in mechanically ventilated ICU patients. Development of the questionnaire was informed by three complementary sources: (1) an earlier retrospective cohort analysis from the same centre, which examined adverse events and risk contexts during intensive care HBOT sessions [3]; (2) local peri-HBOT workflow analysis in a highly specialised hyperbaric facility with extensive experience in the management of critically ill patients; and (3) available literature addressing critically ill patient transport, HBOT-specific equipment constraints, and checklist-based safety support.

The questionnaire items addressed key steps in the local peri-HBOT pathway, including pre-session preparation, transfer and chamber readiness, equipment adaptation, airway-related precautions, infusion and monitoring management, and post-HBOT restoration of standard ICU support. The draft instrument was reviewed internally by the author team for clinical relevance, clarity, apparent face and content relevance, and consistency with established local practice. No formal cognitive interviewing, pre-use pilot testing, psychometric validation, inter-rater reliability assessment, external expert panel, or Delphi consensus process was performed before survey administration. Therefore, this internal review should not be interpreted as formal validation. The full questionnaire, including the original version used in the study and an English translation, is provided as Supplementary File S1 for transparency and critical appraisal.

### 2.5. Data Collection

Each respondent completed the questionnaire once. Respondents were asked to indicate whether they had observed each listed task being omitted, delayed, incompletely performed, or temporarily interrupted at least once during the preceding month in relation to the preparation of mechanically ventilated patients for HBOT or their post-HBOT management and stabilisation. They were not asked to estimate how many times a given omission had occurred during that period. For the purpose of this study, an omission was defined operationally as a respondent-perceived failure to perform or complete a listed safety-related step when it was considered indicated within the local peri-HBOT process. The questionnaire was therefore designed to capture the occurrence of staff-observed omissions at the respondent level rather than event-level or patient-level frequencies; it did not record the number of times a given omission occurred, the number of patients involved, or the specific HBOT session to which the omission referred. To reduce reporting inaccuracy, the questionnaire used a short one-month recall window, anonymous completion, and concrete task wording reflecting the local peri-HBOT workflow. However, no direct observational audit, event log, electronic documentation review, or independent source verification was performed. These design features may have reduced some reporting barriers but could not eliminate recall bias, salience bias, social desirability bias, duplicate reporting, or misclassification of delayed, incomplete, or corrected actions. Data were collected between 1 and 31 May 2024, and all returned questionnaires were included in the analysis.

### 2.6. Data Analysis

Data were analysed descriptively. All analyses were performed at the respondent level. For each checklist item, the numerator was the number of respondents who reported having observed that omission at least once during the preceding month, and the denominator was the total number of respondents (*n* = 19).

Given the small sample size (*n* = 19) and the dichotomous nature of the variables, proportions of reported omissions were summarised using counts, percentages, and Wilson 95% confidence intervals. No inferential comparisons were performed between the pre-HBOT and post-HBOT phases, because these phases involve partially different clinical tasks and the study was designed as an exploratory respondent-level survey rather than a paired before/after analysis of individual events. Accordingly, the reported percentages and confidence intervals should be interpreted as respondent-level descriptive summaries only and should not be treated as estimates of true omission prevalence or as patient-level, session-level, or transfer-level event rates.

All analyses were performed using the R statistical environment version 4.3.3 with the following R packages: sjPlot, performance, report, gtsummary, MASS, and ggplot2.

### 2.7. Checklist Development

The prototype checklist was developed as a pragmatic, context-specific bedside tool in a stepwise process. First, the scope of checklist development was informed by an earlier retrospective cohort analysis conducted at the same centre, which examined 176 intensive care patients treated with HBOT between 2013 and 2023 and identified major clinical contexts associated with adverse events during transport and HBOT sessions [3]. Second, the authors mapped the actual local peri-HBOT workflow at the Hyperbaric Centre, including pre-session bedside preparation, immediate transfer and chamber-readiness tasks, and post-session restoration of standard ICU support [2,3]. Third, an initial pool of candidate checklist items was compiled from three sources: (1) items represented in the study questionnaire (Supplementary File S1), (2) clinically or technically relevant tasks identified during local workflow review, and (3) actions supported by the literature on critically ill patient transport, HBOT care, and checklist-based safety support [2,5,6,7,11,16]. Fourth, candidate items were retained in the prototype if they fulfilled at least one of the following criteria: they represented a required step in the routine local peri-HBOT workflow; they corresponded to an omission reported by respondents in the survey; or they were judged by the authors to represent a clinically important or technically necessary step in the hyperbaric environment despite lower reported frequency [2,3,7]. This applied in particular to items related to airway preparedness, exchange of cuff medium in the endotracheal tube, removal of hazardous materials, use of HBOT-compatible equipment, and restoration of standard ICU therapies after HBOT (Supplementary File S1). Fifth, the retained items were organised into sequential phases reflecting the actual local workflow and phrased as short, action-oriented checks intended to preserve bedside usability and support team communication [11,16,17]. The checklist was developed de novo rather than by direct adaptation of a generic intrahospital transport checklist because no validated instrument specific to peri-HBOT management of mechanically ventilated ICU patients was identified, and existing transport templates do not fully address several tasks central to the local HBOT pathway, including cuff-medium exchange, chamber-specific equipment adaptation, environmental safety constraints, and post-session re-establishment of ICU support [2,5,6]. The resulting checklist prototype, provided in full as a high-resolution Supplementary File S2, should therefore be understood as a context-specific prototype derived from prior risk identification, local workflow analysis, literature review, and exploratory survey findings, rather than as a universal checklist for all ICU transfers. Formal external validation of the checklist was not part of the present pilot study. No formal operational risk assessment of ICU-to-HBOT transport was performed locally before or alongside development of the present checklist; instead, the prototype was informed by retrospective clinical risk identification, local workflow review, literature review, and exploratory survey findings.

A checklist-item derivation matrix is provided as Supplementary Table S1 to document how each retained prototype item relates to the corresponding survey item, local workflow rationale, literature/HBOT rationale, reason for retention, and item applicability.

### 2.8. Ethical Considerations

The study was approved by the Independent Bioethics Committee for Scientific Research at the Medical University of Gdańsk (approval no. KB/52/2024; 28 March 2024). Participation was entirely voluntary. All eligible staff members were provided with information regarding the study objectives and procedures prior to participation. Informed consent for participation was obtained from all respondents. Participants were free to decline participation at any time without providing a reason and without any consequences. The survey was anonymous; no directly identifying information (e.g., names, personal identification numbers, or contact details) was collected. No artificial intelligence tools were used in the design, conduct, or analysis of the study.

## 3. Results

### 3.1. Characteristics of the Study Group

A total of 19 healthcare professionals participated in the study, including 13 women and 6 men. The sample consisted of 13 nurses and 6 physicians employed at the Hyperbaric Centre (HC). Most respondents (89.47%) reported previous experience using checklists, and more than half (63.16%) had over 10 years of professional experience at the HC. The reported percentages in subsequent analyses are based on the number of respondents (*n* = 19), not on the number of patients, HBOT sessions, or transfer episodes. The detailed characteristics of the study group are presented in Table 1.

### 3.2. Respondent-Reported Observations Before HBOT

Table 2 summarises respondent-reported observations during pre-HBOT preparation. Percentages represent the proportion of respondents who indicated that they had observed a given task being omitted, delayed, incompletely performed, or temporarily interrupted at least once during the preceding month. Before HBOT, the most frequently reported observations concerned airway rescue readiness and transfer/chamber preparation. The most prominent item was preparation of the intubation set for transfer (14/19, 73.7%; 95% CI: 51.2–88.2), followed by disconnection of the anti-decubitus mattress pump (8/19, 42.1%; 95% CI: 23.1–63.7), removal or replacement of hazardous materials from the patient’s bed (7/19, 36.8%; 95% CI: 19.1–59.0), and preparation of the self-inflating bag (6/19, 31.6%; 95% CI: 15.4–54.0). Other items were reported by fewer respondents or were not reported during the study month. Confidence intervals were wide for several items, reflecting the limited precision associated with the small sample size.

### 3.3. Respondent-Reported Observations After HBOT

Table 3 summarises respondent-reported observations during post-HBOT stabilisation. Percentages represent the proportion of respondents who indicated that they had observed a given task being omitted, delayed, incompletely performed, or temporarily interrupted at least once during the preceding month. After HBOT, the most frequently reported observations concerned restoration of standard ICU support after treatment. The most prominent item was replacement of fluid with air in the endotracheal tube cuff (11/19, 57.9%; 95% CI: 36.3–76.9), followed by reconnection of the anti-decubitus mattress pump (10/19, 52.6%; 95% CI: 31.7–72.7). Reconnection of capnography monitoring, replacement of supportive items or materials on the patient’s bed, and reconnection of intravenous infusions interrupted for HBOT were each reported by 5/19 respondents (26.3%; 95% CI: 11.8–48.8). Other items were reported by fewer respondents or were not reported during the study month. As in the pre-HBOT phase, confidence intervals were wide for several items because of the small sample size.

## 4. Discussion

Overall, this single-centre pilot survey generated respondent-level workflow signals rather than verified process failures, omission prevalence, or patient-level event rates. For interpretation, these signals can be grouped into two domains: pre-HBOT airway, transfer, and chamber-readiness tasks, and post-HBOT restoration of standard ICU support after intentional or procedural adaptations during treatment (Table 2 and Table 3).

Most respondents were experienced clinicians with prior checklist exposure, which may support future usability testing of the preliminary peri-HBOT checklist prototype in this setting [18]. However, feasibility, acceptability, compliance, and effectiveness were not evaluated in the present study.

These patterns overlap with the broader literature on the transfer of critically ill patients beyond HBOT-specific care. Respiratory and cardiovascular events are repeatedly reported among important complications during intra-hospital transport of critically ill patients [19], and recent ICU transfer guidance emphasises pre-transfer planning, airway and ventilation readiness, monitoring continuity, equipment preparation, medication availability, documentation, and clear team roles [20,21]. Transport-checklist studies similarly suggest that structured tools can improve compliance with transport-safety requirements and reduce omissions in preparation processes [22,23]. Therefore, the present findings should not be interpreted as indicating an entirely unique HBOT problem. Rather, they show that several generic ICU transfer-safety vulnerabilities may reappear within the peri-HBOT pathway, where they are combined with additional chamber-specific technical and environmental requirements.

The airway-readiness signal observed before HBOT should be interpreted in the context of local workflow and the historical organisation of intra-departmental transfers at the study centre. For many years, transfer to the hyperbaric chamber was performed entirely within the department, and the chamber was previously located on the same floor as the ICU. Under these circumstances, teams may not have routinely carried a full airway kit for every transfer. This historical practice should not be interpreted as evidence of poor-quality care, but as a reflection of a short, familiar, intra-departmental pathway with immediate access to the same trained hyperbaric-ICU team. However, broader critical-care transfer literature supports the principle that airway rescue preparedness and backup ventilation should be explicitly verified before movement of mechanically ventilated patients [19,20,21]. In this respect, the airway-related observations support written confirmation of high-consequence readiness tasks even in short, familiar intra-departmental transfer pathways [23].

At the same time, peri-HBOT management differs from conventional transfer to imaging, operating theatres, or other hospital departments because several tasks are imposed by the hyperbaric environment itself. Removal or replacement of hazardous materials was retained as a checklist item because chamber-safety guidance and local HBOT workflow treat material compatibility as an essential pre-session requirement [1,7]. HBOT-specific equipment constraints also affect infusion-device management and other electrically powered or pressure-sensitive devices [7,24]. Similarly, cuff-medium management is a hyperbaric-specific technical issue, because endotracheal tube cuff pressure and cuff-medium behaviour during HBOT require deliberate management in mechanically ventilated patients [2,25]. These requirements are not usually central elements of generic intrahospital transport checklists [5,21,23]. This supports the rationale for a dedicated peri-HBOT checklist prototype that combines standard ICU transfer-safety checks with chamber-specific preparation and equipment-adaptation steps.

The post-HBOT restoration signal is relevant to checklist design because peri-HBOT care involves deliberate, protocol-driven adaptation of the patient’s environment and equipment before treatment, followed by restoration of standard ICU support after treatment. In particular, replacement of fluid with air in the endotracheal tube cuff was retained as a post-HBOT restoration check because cuff-medium management is an HBOT-specific technical requirement in mechanically ventilated patients [2,25]. The present survey did not assess whether reported observations were associated with airway complications or other patient-level outcomes. Respondent-reported observations involving reconnection of the anti-decubitus mattress pump and interrupted infusions identify post-session return-to-bed steps that may warrant procedural standardisation. This post-session dimension also distinguishes the proposed tool from many generic transport checklists, which tend to focus primarily on pre-transfer readiness, monitoring, escort, equipment, and handover rather than on systematic reversal of procedure-specific adaptations after return to the ICU [5,21,23]. These findings therefore support consideration of explicit post-HBOT stabilisation items in the locally derived checklist prototype alongside transfer- and chamber-readiness items.

Several checklist items had no respondent-reported observations during the study month, including endotracheal tube fixation checks and selected equipment-adaptation or restoration steps. These zero values should not be interpreted as evidence that risk was absent or that compliance was complete. In a respondent-level survey, they may reflect strong local standardisation, limited applicability of a given item during the one-month recall period, absence of patients requiring that specific step, or incomplete recall. These items were retained in the prototype when they represented mandatory local peri-HBOT workflow checks or low-frequency steps for which omission could plausibly have clinically important consequences. Accordingly, future versions of the checklist should include a clear “not applicable” option and should be tested prospectively to determine item applicability, completion burden, and task ownership in routine use. Item-specific applicability considerations for the retained prototype components are summarised in Supplementary Table S1.

A decade ago, a checklist was proposed as one preventive tool to improve the safety of transporting critically ill patients to hyperbaric chambers [4]; however, published reports on its use remain limited. Bosco et al. described checklist-supported preparation during transfer to an external hyperbaric facility and suggested that comprehensive preparation may reduce transport complexity and improve safety [6]. Preininger also described structured preparation forms as a way to streamline preparation of ICU patients for HBOT and support nursing workflow [26]. Previous safety work from the HC examined adverse events and risk contexts during intensive care HBOT sessions [3]. The present pilot study extends this local safety work by shifting attention from adverse-event outcomes to respondent-level observations of preparedness and restoration vulnerabilities. Therefore, the aim was not to reproduce a generic transport template, but to construct a local prototype that integrates general transfer-safety elements with HBOT-specific preparation and post-session restoration steps.

These findings should be interpreted through a human-factors and systems-safety lens, in which patient safety is shaped by interactions between clinicians, tasks, tools and technologies, organisational routines, and the clinical environment [10]. The present survey did not demonstrate that a checklist will improve outcomes; rather, it identified points in the local work system where reliance on memory, implicit task allocation, equipment transitions, and post-session restoration steps may create vulnerability [10,11,14]. In our centre, many peri-HBOT tasks have traditionally been performed using a cognitive, memory-based sequence of checks rather than a written tool. Checklist literature describes checklists as cognitive aids intended to ensure completion of task components, promote adherence to best practice, and prevent errors of omission; it also indicates that memory-based or poorly usable checklist execution may reduce completion reliability [11,14]. Formalising this shared practice into the context-specific prototype provided in Supplementary File S2 may therefore be a plausible strategy for improving consistency under time pressure, but this remains a hypothesis requiring prospective evaluation rather than a finding of the present study [10,11,13].

Accordingly, the proposed checklist should not be treated as a stand-alone intervention. Its potential value will depend on how well it fits the actual peri-HBOT workflow, whether responsibility for completing or verifying each item is explicit, whether end users perceive the items as relevant and non-redundant, and whether implementation is supported by feedback, local adaptation, and prospective monitoring of adherence [10,11,13,14]. This interpretation is consistent with recent checklist implementation evidence showing that dynamic, locally responsive implementation strategies can achieve better checklist compliance than static implementation, while ambiguity, redundancy, time pressure, and limited team buy-in may remain important barriers [13]. Although recent ICU checklist literature suggests potential benefits for selected clinical and process-of-care outcomes, the present study was not designed to evaluate checklist effectiveness, patient outcomes, or adverse-event reduction [11,12]. The survey should therefore be interpreted as an exploratory risk-identification exercise, and the resulting context-specific checklist (Supplementary File S2) as one practical component of a broader local safety framework rather than a stand-alone solution [10,11,13].

The transferability of this prototype is therefore strongly influenced by the organisational model in which it was developed. In the present centre, the ICU and hyperbaric chamber are located within the same building, patients are already accommodated on hyperbaric-adapted beds, transfer is short and intra-departmental, and care is maintained by the same trained hyperbaric-ICU team throughout the peri-HBOT pathway [3]. Internationally, HBOT delivery for critically ill patients may follow different organisational models; for example, some patients may be transported to external or stand-alone hyperbaric facilities, transferred over longer distances, or handed over between referring ICU, transport, and chamber teams [6,21]. These differences may change the relative importance of checklist domains: centres using longer or inter-hospital transfer pathways may need more detailed items on transport duration, escort composition, oxygen and power autonomy, emergency medications, documentation, and structured handover [5,20,21], whereas integrated ICU-HBOT pathways may place greater emphasis on chamber readiness, equipment adaptation, and post-session restoration of standard ICU support. Nevertheless, several domains in the present prototype address requirements inherent to preparing mechanically ventilated ICU patients for HBOT, including chamber material compatibility, HBOT-compatible equipment, airway and ventilation readiness, pressure-related cuff-medium management, infusion-line safety, monitoring continuity, and restoration of standard ICU support after the session [1,2,7,21,24,25]. The prototype should therefore be regarded as a locally derived peri-HBOT checklist framework that highlights HBOT-specific safety domains and may inform local adaptation, not as a ready-to-use international standard.

### 4.1. Strength and Limitations of the Work

This study has several strengths. First, it addressed a highly specific and clinically important area of nursing and interdisciplinary practice that remains underreported in the literature, namely, the preparation, intra-departmental transfer, and post-treatment stabilisation of mechanically ventilated intensive care patients undergoing HBOT. Second, the study was conducted in a highly specialised hyperbaric centre with longstanding experience in the management of critically ill patients requiring HBOT. This setting allowed the survey items and checklist domains to reflect real peri-HBOT workflow rather than a theoretical transfer model. The present work also builds on previous safety research from the same centre, in which adverse events and risk contexts during intensive care HBOT were retrospectively analysed. This continuity strengthens the clinical rationale for focusing on peri-procedural preparedness and post-session restoration tasks. Third, the survey included both nurses and physicians directly involved in peri-HBOT care.

The study also has important limitations. First, it was a single-centre pilot survey conducted in a distinctive organisational model. At the study centre, mechanically ventilated ICU patients undergoing HBOT are transferred within the same building and cared for by the same trained hyperbaric-ICU team before, during, and after treatment. These features increase the clinical specificity of the findings but limit transferability to centres using remote, external, inter-hospital, or handover-based HBOT pathways. Therefore, the checklist should not be assumed to be directly generalisable without local adaptation and external validation. Second, the sample was small (*n* = 19), resulting in wide confidence intervals and limited precision. Third, the data were based on retrospective respondent recall over the preceding month rather than direct observation, prospective process measurement, electronic documentation, or event-based reporting. Recall bias, salience bias, and social desirability bias therefore cannot be excluded. The one-month recall window may have reduced long-term recall error but could not eliminate inaccurate or selective reporting. Fourth, the questionnaire was locally developed and was not subjected to formal psychometric validation, reliability testing, multicentre pilot testing, or external expert consensus. Although the instrument was reviewed by the author team for clinical relevance, clarity, apparent face/content relevance, and alignment with local practice, this internal review does not constitute formal validation. Fifth, the operational category of “omission” was broad and respondent-perceived. The survey did not distinguish between complete non-performance, delayed performance, incomplete performance, temporary interruption followed by correction, or a task judged unnecessary in a specific clinical situation. Item applicability may also have varied between patients and HBOT sessions, particularly for tasks related to renal replacement therapy, drainage systems, or selected infusion-management procedures. Sixth, the survey did not collect patient-level, session-level, transfer-level, or event-level identifiers. It was therefore not possible to determine the number of unique patients represented, whether several HBOT sessions involved the same patient, or whether more than one respondent referred to the same observed event. Duplicate reporting may have overestimated the apparent frequency and relative prominence of some reported observations, whereas under-recognition or non-reporting may have underestimated others. Seventh, the study did not include adverse-event data, patient outcomes, direct process auditing, or independent verification of reported observations. Therefore, no causal relationship can be inferred between respondent-reported omissions and patient harm, treatment interruption, transport complications, or clinical outcomes. The findings identify perceived workflow vulnerabilities and candidate targets for standardisation, not confirmed determinants of adverse events. Finally, the checklist developed from this work should be considered a preliminary, locally derived prototype. The study did not test checklist implementation, compliance, usability, feasibility, acceptability, effect on omission rates, or effect on patient safety outcomes. Further prospective evaluation, structured end-user refinement, external expert review, and multicentre validation across different HBOT organisational models are required before the checklist can be recommended for broader use.

### 4.2. Recommendations for Further Research and Validation

Further research should focus on structured evaluation of the prototype checklist developed in this study. The checklist is currently in the final stage of local implementation and end-user refinement at the study centre. This translational pathway includes prospective usability testing, structured stakeholder engagement, and iterative feedback from nurses and physicians directly responsible for peri-HBOT preparation, transfer, chamber-related equipment adaptation, and post-session restoration of standard ICU support.

Because the present study was a single-centre pilot survey, local implementation and refinement should not be interpreted as external validation. After completion of the local implementation and end-user refinement phase, the next validation step should include direct consultation with external HBOT centres and relevant national or international HBOT organisations, together with structured expert review or consensus work before broader multicentre evaluation. Multicentre studies involving different HBOT organisational models are still needed, including centres with remote, inter-hospital, or handover-based transfer pathways. Future research should assess whether checklist use is associated with improved process reliability, reduction in independently observed omissions, continuity of care, transport safety, and, where feasible, patient-safety outcomes. Until such validation is completed, the checklist should be regarded as a locally derived preliminary prototype rather than a broadly generalisable tool.

## 5. Conclusions

This single-centre pilot survey identified staff-reported peri-HBOT workflow vulnerabilities among mechanically ventilated ICU patients, particularly in airway and transfer readiness before HBOT and restoration of standard ICU support after HBOT. The findings are preliminary respondent-level observations and should not be interpreted as verified omission rates, evidence of patient harm, or proof of checklist effectiveness. They may inform further development and prospective evaluation of a locally derived preliminary checklist prototype across different HBOT organisational models.

## Figures and Tables

**Table 1:** Characteristics of the study participants.

Characteristic	Category	*n* (%)
Sex	Female	13 (68.4)
	Male	6 (31.6)
Profession	Physicians	6 (31.6)
	Nurses	13 (68.4)
Work experience in HC	Up to 10 years	7 (36.8)
	Over 10 years	12 (63.2)
Previous experience with checklists	No	2 (10.5)
	Yes	17 (89.5)

*n* (%), number and percentage of respondents; HC, Hyperbaric Centre.

**Table 2:** Respondent-reported observations before HBOT.

Characteristics	*n*/*N* (%)	95% CI
Preparing the intubation set for transfer	14/19(73.68%)	51.2–88.2
Disconnecting the anti-decubitus mattress pump from the anti-decubitus mattress	8/19 (42.11%)	23.1–63.7
Replacement or removal of hazardous materials from the patient’s bed	7/19 (36.84%)	19.1–59
Preparing the self-inflating manual resuscitator bag	6/19 (31.58%)	15.4–54
Replacing air with fluid in the endotracheal tube cuff	4/19 (21.05%)	8.5–43.3
Securing the gastric tube with a decompression bag	3/19 (15.79%)	5.5–37.6
Suctioning of secretions from the respiratory tract and oral cavity	3/19 (15.79%)	5.5–37.6
Checking the presence of air bubbles in the infusion lines	3/19 (15.79%)	5.5–37.6
Connecting capnography monitoring	3/19 (15.79%)	5.5–37.6
Preparing required medications for transport and for HBOT	2/19 (10.53%)	2.9–31.4
Disconnecting unnecessary/unwanted equipment	2/19 (10.53%)	2.9–31.4
Protecting drainage systems with a one-way valve	1/19 (5.26%)	0.9–24.6
ECG, IBP, SpO_2_ monitoring	1/19 (5.26%)	0.9–24.6
Switching infusions to HBOT-dedicated pumps	0/19 (0%)	0–16.8
Disconnecting renal replacement therapy	0/19 (0%)	0–16.8
Switching the patient to a HBOT-dedicated mechanical ventilator	0/19 (0%)	0–16.8
Checking fixation of the endotracheal tube	0/19 (0%)	0–16.8
Other	0/19 (0%)	0–16.8

ECG, electrocardiography; IBP, invasive blood pressure; SpO_2_, peripheral oxygen saturation; *n*/*N* (%), number/total number (percentage) of respondents; 95% CI, 95% Wilson confidence interval; HBOT, Hyperbaric oxygen therapy. Values are respondent-level descriptive summaries. Percentages are based on the total number of respondents (*N* = 19) and indicate the proportion of respondents who reported observing a given task being omitted, delayed, incompletely performed, or temporarily interrupted at least once during the preceding month.

**Table 3:** Respondent-reported observations after HBOT.

Characteristics	*n*/*N* (%)	95% CI
Replacing fluid with air in the endotracheal tube cuff	11/19 (57.89%)	36.3–76.9
Reconnecting the anti-decubitus mattress pump	10/19 (52.63%)	31.7–72.7
Replacing supportive items/materials previously removed from the patient’s bed	5/19 (26.32%)	11.8–48.8
Reconnecting capnography monitoring	5/19 (26.32%)	11.8–48.8
Other (reconnection of infusions interrupted for HBOT)	5/19 (26.32%)	11.8–48.8
Checking the presence of air bubbles in the infusion lines	3/19 (15.79%)	5.5–37.6
ECG, IBP, SpO_2_ monitoring	3/19 (15.79%)	5.5–37.6
Suctioning of secretions from the respiratory tract and oral cavity	1/19 (5.26%)	0.9–24.6
Checking fixation of the endotracheal tube	0/19 (0%)	0–16.8
Securing drains/drainage systems with dedicated equipment	0/19 (0%)	0–16.8
Switching intravenous infusions back to bedside syringe pumps	0/19 (0%)	0–16.8
Reconnecting feeding to the gastric tube	0/19 (0%)	0–16.8
Reconnecting previously disconnected equipment, including renal replacement therapy	0/19 (0%)	0–16.8
Reconnecting the patient to the bedside mechanical ventilator	0/19 (0%)	0–16.8

ECG, electrocardiography; IBP, invasive blood pressure; SpO_2_, peripheral oxygen saturation; *n*/*N* (%), number/total number (percentage) of respondents; 95% CI, 95% Wilson confidence interval; HBOT, Hyperbaric oxygen therapy. Values are respondent-level descriptive summaries. Percentages are based on the total number of respondents (*N* = 19) and indicate the proportion of respondents who reported observing a given task being omitted, delayed, incompletely performed, or temporarily interrupted at least once during the preceding month.

## Data Availability

The datasets generated and/or analysed during the current study are not publicly available due to confidentiality considerations and the small single-centre sample, which may increase the risk of deductive disclosure. De-identified, item-level data may be made available by the corresponding author upon reasonable request for scientifically justified purposes, subject to approval by the relevant institutional authority where required. Data will be shared only to the extent permitted by institutional and ethical requirements. Anonymised study data are stored on a password-protected institutional drive and on a separate password-protected external drive held by the principal investigator. The study questionnaire is available in the Supplementary Materials.

## Supplementary Materials

The following supporting information can be downloaded at: https://www.mdpi.com/article/10.3390/jcm15145418/s1, Supplementary File S1: Study questionnaire in Polish and English translation; Supplementary File S2: Prototype peri-HBOT checklist structured for sequential use during pre-session preparation and transfer readiness, and again after HBOT for restoration of standard ICU support; Supplementary Table S1: Checklist-item derivation matrix for the preliminary peri-HBOT checklist prototype [1,2,3,4,6,7,19,20,21,23,24,25,26,27,28,29,30].

## Abbreviations

The following abbreviations are used in this manuscript:

ATA	Absolute atmospheres
CO	Carbon monoxide
HBOT	Hyperbaric oxygen therapy
HC	Hyperbaric Centre
ICU	Intensive care unit
NSTI	Necrotizing soft tissue infections
