# Supplementary material for: Staff-Reported Peri-Procedural Workflow Vulnerabilities and a Preliminary Checklist Prototype for Mechanically Ventilated Intensive Care Patients Undergoing Hyperbaric Oxygen Therapy: A Single-Centre Pilot Survey
Source: J Clin Med. 2026 Jul 10;15(14):5418. doi: 10.3390/jcm15145418 (PMC13410505; doi:10.3390/jcm15145418)
Supplement: Supplementary file 1 [file jcm-15-05418-s001.zip › Supplementary File S1.pdf]

Szanowni Państwo, zwracam się z uprzejmą prośbą o wypełnienie kwestionariusza ankiety, który pozwoli określić ryzyko zdarzeń niepożądanych podczas transportu pacjenta intensywnej terapii KMHiRM do komory hiperbarycznej i dzięki temu stworzyć proste narzędzie, które pomoże zminimalizować określone ryzyko. Kwestionariusz ten skierowany jest do kadry pielęgniarskiej oraz lekarskiej KMHiRM UCMMiT w Gdyni. Jest on anonimowy i w pełni dobrowolny, a wszystkie umieszczone tutaj informacje posłużą tylko i wyłącznie celom naukowym, przystępując do niego wyrażają Państwo zgodę na jego wypełnienie, które zajmie nie więcej niż 5-10 minut. Projekt posiada pozytywną opinię Niezależnej Komisji Bioetycznej do spraw Badań Naukowych przy Gdańskim Uniwersytecie Medycznym.

Z wyrazami szacunku,

mgr Aneta Miszewska.

- ☐ **Wyrażam świadomą i dobrowolną zgodę na wypełnienie kwestionariusza**

Zawód:

- ☐ Lekarz
- ☐ Pielęgniarka

Płeć:

- ☐ Kobieta
- ☐ Mężczyzna

Staż pracy w KMHiRM UCMMiT w Gdyni:

- ☐ 1-5 lat
- ☐ 6-10 lat
- ☐ 11-20 lat
- ☐ Więcej niż 20 lat

Czy posiadają Państwo doświadczenie pracy z checklistami/listami kontrolnymi:

- ☐ Tak
- ☐ Nie

Proszę zaznaczyć (X) wszystkie **przeoczone** czynności, z którymi spotkali się Państwo w ciągu ostatniego miesiąca w trakcie przygotowywania pacjenta intensywnej terapii **do transportu do komory hiperbarycznej**.

- ☐ Wymiana/usunięcie niebezpiecznych materiałów z łóżka pacjenta.
- ☐ Odłączenie pompy od materaca p/odleżynowego.
- ☐ Zabezpieczenie sondy żołądkowej workiem odbarczającym.
- ☐ Odessanie wydzieliny z dróg oddechowych i jamy ustnej.
- ☐ Kontrola umocowania rurki intubacyjnej.
- ☐ Wymiana w mankiecie uszczelniającym rurki intubacyjnej powietrza na płyn.

- Zabezpieczenie drenaży/drenów zastawką jednokierunkową.
- Przygotowanie potrzebnych leków na czas transportu i pobytu w komorze hiperbarycznej.
- Przełączenie wlewów dożylnych do pomp strzykawkowych dedykowanych HBO.
- Skontrolowanie obecności pęcherzyków powietrza w przewodach infuzyjnych.
- Monitorowanie EKG, IBP, SpO2.
- Odłączenie zbędnego/niepożądanego sprzętu.
- Odłączenie terapii nerkozastępczej.
- Przygotowanie zestawu do intubacji na czas transportu.
- Przygotowanie worka samorozprężalnego/AMBU.
- Przełączenie chorego do respiratora dedykowanego HBO.
- Kapnometria.
- Inne:……

Proszę zaznaczyć (X) wszystkie **przeoczone** czynności, z którymi spotkali się Państwo w ciągu ostatniego miesiąca **po powrocie pacjenta intensywnej terapii z komory hiperbarycznej**.

- Ponowne zabezpieczenie pacjenta w udogodnienia/ materiały, które leżały na łóżku chorego.
- Podłączenie pompy od materaca p/odleżynowego.
- Podłączenie żywienia do sondy żołądkowej.
- Odessanie wydzieliny z dróg oddechowych i jamy ustnej.
- Kontrola umocowania rurki intubacyjnej.
- Wymiana w mankiecie uszczelniającym rurki intubacyjnej płynu na powietrze.
- Zabezpieczenie drenów/drenaży.
- Przełączenie wlewów dożylnych do przyłóżkowych pomp strzykawkowych.
- Skontrolowanie obecności pęcherzyków powietrza w przewodach infuzyjnych.
- Monitorowanie EKG, IBP, SpO2.
- Podłączenie wcześniej odłączonego sprzętu, w tym terapii nerkozastępczej.
- Podłączenie chorego do respiratora przyłóżkowego.
- Podłączenie kapnometrii.
- Inne:……

Obowiązek informacyjny wobec uczestników projektów przeprowadzanych w Gdańskim Uniwersytecie Medycznym

Zgodnie z art. 13 ogólnego rozporządzenia o ochronie danych osobowych z dnia 27 kwietnia 2016 r. informuje, że:

1) Administratorem Pani/Pana Danych Osobowych jest Gdański Uniwersytet Medyczny z siedzibą ul. M. Skłodowskiej-Curie 3A, 80-210 Gdańsk, reprezentowany przez Rektora,

2) Kontakt do Inspektora Ochrony Danych w Gdańskim Uniwersytecie Medycznym, adres email: iod@gumed.edu.pl,

3) Pani/Pana dane osobowe przetwarzane będą w celu realizacji Projektu: „Bezpieczny transport pacjentów IT na sesję w komorze hiperbarycznej. Identyfikacja ryzyka zdarzeń niepożądanych." oraz przyszłych badań naukowych odnośnie zdrowia, w Uczelni na podstawie art. 6 ust. 1 lit. a oraz art. 9 ust. 2 pkt j ogólnego rozporządzenia o ochronie danych osobowych z dnia 27 kwietnia 2016 r.,

4) Pani/Pana dane osobowe mogą być ujawniane wyłącznie osobom upoważnionym u administratora do przetwarzania danych osobowych, podmiotom przetwarzającym na mocy umowy powierzenia oraz innym podmiotom upoważnionym na podstawie przepisów prawa,

5) Pani/Pana dane osobowe przechowywane będą wyłącznie przez okres niezbędny do realizacji badań,

6) posiada Pani/Pan prawo dostępu do treści swoich danych, prawo do ich sprostowania, usunięcia, ograniczenia przetwarzania, prawo do przenoszenia danych, prawo do wniesienia sprzeciwu,

7) posiada Pani/Pan prawo do cofnięcia zgody na przetwarzanie danych osobowych w dowolnym momencie,

8) posiada Pani/Pan prawo wniesienia skargi do Prezesa Urzędu Ochrony Danych Osobowych, gdy uzasadnione jest, że Pani/Pana dane osobowe przetwarzane są przez administratora niezgodnie z ogólnym rozporządzeniem o ochronie danych osobowych z dnia 27 kwietnia 2016 r.,

9) podanie danych osobowych jest dobrowolne, ale niezbędne do realizacji Projektu.

Dear Colleagues,

I would kindly ask you to complete this questionnaire, which is intended to help identify the risk of adverse events during the transfer of an intensive care patient from the Department of Hyperbaric Medicine and Sea Rescue to the hyperbaric chamber and thereby support the development of a simple tool to help minimize this risk. This questionnaire is addressed to the nursing and medical staff of the Department of Hyperbaric Medicine and Sea Rescue, University Centre for Maritime and Tropical Medicine in Gdynia. It is anonymous and entirely voluntary, and all information provided will be used solely for scientific purposes. By proceeding, you give your informed consent to complete the questionnaire, which should take no more than 5–10 minutes. The project has received a positive opinion from the Independent Bioethics Committee for Scientific Research at the Medical University of Gdańsk.

Sincerely,

Aneta Miszewska, MSc

☐ I give my informed and voluntary consent to complete the questionnaire.

Profession:

☐ Physician

☐ Nurse

Sex:

☐ Female

☐ Male

Length of employment at the Department of Hyperbaric Medicine and Sea Rescue, University Centre for Maritime and Tropical Medicine in Gdynia:

☐ 1–5 years

☐ 6–10 years

☐ 11–20 years

☐ More than 20 years

Do you have experience working with checklists / control lists?

☐ Yes

☐ No

Please mark (X) all omitted steps that you have encountered during the preceding month while preparing an intensive care patient for transfer to the hyperbaric chamber.

☐ Replacement or removal of hazardous materials from the patient's bed

☐ Disconnecting the anti-decubitus mattress pump from the anti-decubitus mattress

☐ Securing the gastric tube with a decompression bag

☐ Suctioning secretions from the respiratory tract and oral cavity

☐ Checking fixation of the endotracheal tube

☐ Replacing air with fluid in the endotracheal tube cuff

☐ Protecting drainage systems with one-way valve

☐ Preparing required medications for transport and for HBOT

☐ Switching infusions to HBOT-dedicated pumps

☐ Checking the presence of air bubbles in the infusion lines

☐ ECG, IBP, SpO_2 monitoring

☐ Disconnecting unnecessary / unwanted equipment

☐ Disconnecting renal replacement therapy

☐ Preparing the intubation set for transfer

☐ Preparing the self-inflating bag / AMBU bag

☐ Switching the patient to an HBOT-dedicated mechanical ventilator

☐ Connecting capnometry monitoring

☐ Other: ………

………

………

Please mark (X) all omitted steps that you have encountered during the preceding month after the return of an intensive care patient from the hyperbaric chamber.

☐ Replacing supportive items/materials previously removed from the patient’s bed

☐ Reconnecting the anti-decubitus mattress pump

☐ Reconnecting feeding to the gastric tube

☐ Suctioning secretions from the respiratory tract and oral cavity

☐ Checking fixation of the endotracheal tube

☐ Replacing fluid with air in the endotracheal tube cuff

☐ Securing drains / drainage systems with dedicated equipment

☐ Switching intravenous infusions back to bedside syringe pumps

☐ Checking the presence of air bubbles in the infusion lines

☐ ECG, IBP, SpO_2 monitoring

☐ Reconnecting previously disconnected equipment, including renal replacement therapy

☐ Reconnecting the patient to the bedside mechanical ventilator

☐ Reconnecting capnometry

☐ Other: ………

………

………

Information clause for participants in projects conducted at the Medical University of Gdańsk

Pursuant to Article 13 of the General Data Protection Regulation of 27 April 2016, you are informed that:

The controller of your personal data is the Medical University of Gdańsk, with its registered office at ul. M. Skłodowskiej-Curie 3A, 80-210 Gdańsk, represented by the Rector.

The Data Protection Officer at the Medical University of Gdańsk may be contacted at: iod@gumed.edu.pl

Your personal data will be processed for the purpose of conducting the project: “Safe transfer of intensive care patients for a session in the hyperbaric chamber. Identification of adverse event risk factors” and future scientific research concerning health at the University, pursuant to Article 6(1)(a) and Article 9(2)(j) of the General Data Protection Regulation of 27 April 2016.

Your personal data may be disclosed only to persons authorized by the controller to process personal data, entities processing data under a data processing agreement, and other entities authorized under applicable law.

Your personal data will be stored only for the period necessary to conduct the research.

You have the right to access your data, rectify it, erase it, restrict its processing, transfer it, and object to its processing.

You have the right to withdraw your consent to the processing of personal data at any time.

You have the right to lodge a complaint with the President of the Personal Data Protection Office if you believe that your personal data are being processed by the controller in breach of the General Data Protection Regulation of 27 April 2016.

Providing personal data is voluntary, but necessary for participation in the project.
