# Supplementary material for: Staff-Reported Peri-Procedural Workflow Vulnerabilities and a Preliminary Checklist Prototype for Mechanically Ventilated Intensive Care Patients Undergoing Hyperbaric Oxygen Therapy: A Single-Centre Pilot Survey
Source: J Clin Med. 2026 Jul 10;15(14):5418. doi: 10.3390/jcm15145418 (PMC13410505; doi:10.3390/jcm15145418)
Supplement: Supplementary file 1 [file jcm-15-05418-s001.zip › Supplementary File S2.pdf]

pre-HBOT/post-HBOT ✓-performed ✗-not performed ☐-not applicable

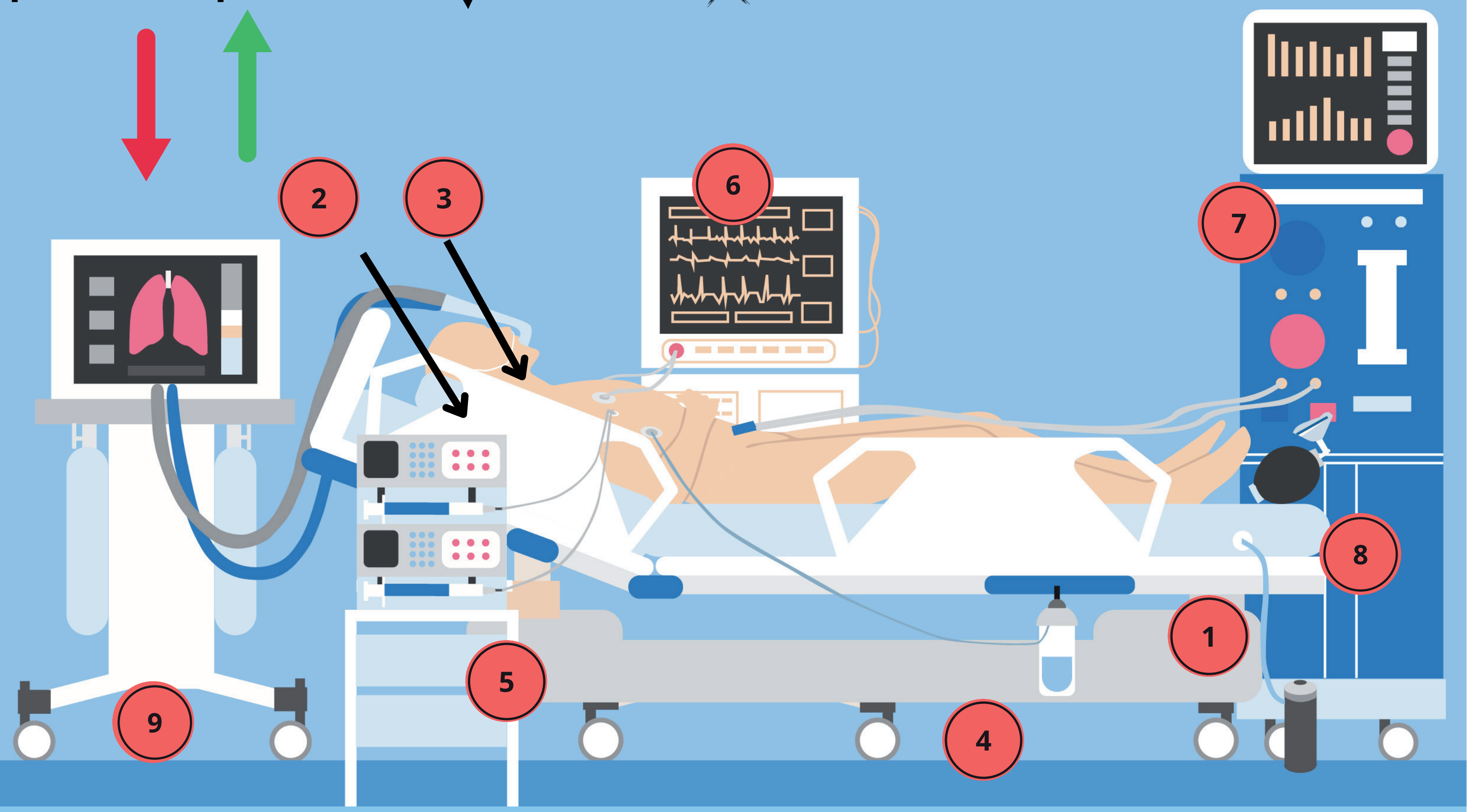

Category	Item
1 Hospital bed	☐ Removal or replacement of hazardous materials
	☐ Disconnecting the electric pump from the anti-decubitus mattress
2 Gastric tube	☐ Securing the gastric tube with a decompression bag
3 Airway	☐ Suctioning secretions from the airway and oral cavity
	☐ Checking fixation of the endotracheal tube
	☐ Replacing air with fluid in the endotracheal tube cuff
4 Drainage systems	☐ Securing drainage systems with one-way valves
5 Medication management	☐ Preparing required medications
	☐ Switching infusions to HBOT-dedicated pumps
	☐ Checking for air bubbles in infusion lines
6 Monitoring	☐ Ensuring ECG, invasive blood pressure (IBP), and SpO_2 monitoring
7 Limited equipment	☐ Disconnecting unnecessary or non-essential equipment
	☐ Disconnecting renal replacement therapy (RRT)
8 Safety	☐ Preparing the intubation set for transport
	☐ Preparing the self-inflating manual resuscitator bag
9 Ventilator	☐ Switching the patient to an HBOT-dedicated mechanical ventilator
	☐ Connecting capnography monitoring

Prototype peri-HBOT checklist structured for sequential use during pre-session preparation and transfer readiness, and again after HBOT for restoration of standard ICU support. Abbreviations: HBOT, hyperbaric oxygen therapy; ECG, electrocardiography; IBP, invasive blood pressure; SpO_2, peripheral oxygen saturation; RRT, renal replacement therapy).
