# Supplementary material for: Staff-Reported Peri-Procedural Workflow Vulnerabilities and a Preliminary Checklist Prototype for Mechanically Ventilated Intensive Care Patients Undergoing Hyperbaric Oxygen Therapy: A Single-Centre Pilot Survey
Source: J Clin Med. 2026 Jul 10;15(14):5418. doi: 10.3390/jcm15145418 (PMC13410505; doi:10.3390/jcm15145418)
Supplement: Supplementary file 1 [file jcm-15-05418-s001.zip › Supplementary Table S1.pdf]

Supplementary Table S1. Checklist-item derivation matrix for the preliminary peri-HBOT checklist prototype

Note. This matrix documents how each checklist component was retained in the preliminary prototype. Reported values are respondent-level descriptive findings from 19 staff members and do not represent verified event counts, patient-level rates, session-level rates, omission prevalence, adverse events, or evidence of checklist effectiveness. Reference numbers correspond to the final main manuscript reference list.

Checklist item	Phase	Corresponding survey item	Reported by respondents n/N (%)	Local workflow rationale	Literature/HBOT rationale	Reason retained	Applicability notes
Removal or replacement of hazardous materials from the patient's bed	pre-HBOT (before HBOT)	Replacement or removal of hazardous materials from the patient's bed	7/19 (36.84%)	Before transfer into the hyperbaric pathway, patient-bed materials must be checked so that non-essential, unsafe, or incompatible items are removed or replaced according to local chamber-safety practice.	HBOT guidance and hyperbaric equipment literature emphasise chamber/fire safety, material compatibility, and restrictions on equipment introduced into the chamber; ICU-transfer guidance supports equipment and environment checks before movement [1,2].	Reported survey signal; HBOT-specific environmental constraint; local workflow requirement; plausible consequence if missed.	Mark N/A only when no removable or replaceable items are present. This item is a workflow check, not evidence that an actual fire or material-safety event occurred.
Disconnecting the electric pump from the anti-decubitus mattress	pre-HBOT (before HBOT)	Disconnecting the anti-decubitus mattress pump from the anti-decubitus mattress	8/19 (42.11%)	The patient remains on a hyperbaric-adapted bed; the electric mattress pump must be disconnected before entering the HBOT pathway according to local equipment practice.	Hyperbaric equipment literature emphasises chamber-compatible devices and comprehensive assessment or limitation of electrically powered equipment; [1,7].	Frequent respondent-reported vulnerability; HBOT-specific equipment constraint; required local workflow step.	Applicable when the anti-decubitus mattress pump is in use. If an alternative pressure-area-care system is used, adapt locally.
Securing the gastric tube with a decompression bag	pre-HBOT (before HBOT)	Securing the gastric tube with a decompression bag	3/19 (15.79%)	Gastric decompression/securement is part of local preparation to reduce distension- or pressure-related issues and avoid uncontrolled tube management during transfer/session.	Critical-care transport literature supports securing tubes, drains, and other attachments before movement; HBOT position statements and equipment literature add pressure-related and gas-containing device considerations [1,2,27].	Survey signal; local workflow requirement; potential clinical consequence if omitted.	Applicable only when a gastric tube is present and decompression is indicated. Record N/A when absent or clinically contraindicated.
Suctioning secretions from the airway and oral cavity	pre-HBOT (before HBOT)	Suctioning of secretions from the respiratory tract and oral cavity	3/19 (15.79%)	Airway toilet is checked before transfer because airway access and emergency interventions may be more constrained during chamber-related care.	Critical-care transport guidance and reviews emphasise airway assessment, optimisation, and emergency preparedness before moving ventilated patients; HBOT guidance stresses trained staff and appropriate equipment for critically ill patients [2,4,19,27].	Survey signal; local airway-preparation step; plausible clinical consequence if omitted.	Applicable according to clinical assessment.
Checking fixation of the endotracheal tube	pre-HBOT (before HBOT)	Checking fixation of the endotracheal tube	0/19 (0%)	Tube fixation is checked before transfer to minimise risk of displacement during movement between ICU and chamber.	Critical-care transport guidance identifies airway security as a core pre-transfer requirement; mechanically ventilated HBOT patients require appropriate equipment, monitoring, and trained personnel [2,4,20,27].	Local mandatory airway-related workflow check; retained because omission could plausibly have clinically important consequences; retained despite zero respondent-reported observations.	Zero reports do not prove perfect compliance. Applicable to all orally/nasally intubated patients; adapt for tracheostomy where relevant.
Replacing air with fluid in the endotracheal tube cuff	pre-HBOT (before HBOT)	Replacing air with fluid in the endotracheal tube cuff	4/19 (21.05%)	Cuff-medium exchange before compression is a local HBOT-specific step intended to avoid pressure-related cuff-volume changes during HBOT.	HBOT-specific sources discuss pressure effects and management of gas-containing components [4,26,27].	Survey signal; HBOT-specific technical requirement; plausible airway-management consequence if omitted.	Applicable to cuffed endotracheal or tracheostomy tubes when local protocol requires fluid-filled cuffs during HBOT.
Securing drainage systems with one-way valves	pre-HBOT (before HBOT)	Protecting drainage systems with a one-way valve	1/19 (5.26%)	Drainage systems must be secured before transfer/session to prevent disconnection, or pressure-related malfunction according to local practice.	Critical-care transport literature supports securing tubes and drains before movement; hyperbaric equipment literature addresses drainage systems and pressure-related device behaviour in the hyperbaric environment [20,21].	Local workflow requirement; plausible clinical consequence if omitted; survey signal present, although infrequent.	Applicable only when drainage systems are present. Device-specific local rules should override generic wording.
Preparing required medications	pre-HBOT (before HBOT)	Preparing required medications for transport and for HBOT	2/19 (10.53%)	Medication availability is checked before the patient leaves the ICU area so that time-critical or rescue treatment is not delayed during transfer or chamber preparation.	Critical-care transfer guidance supports pre-transfer planning of medications and emergency drugs; HBOT care may further restrict immediate access to usual ICU medication infrastructure during chamber-related care [20,21,27].	Survey signal; standard transfer-readiness requirement; plausible treatment-delay consequence if omitted.	Applicable to medications required by the patient's condition and local protocol; avoid unnecessary medication transfer if not clinically required.
Switching infusions to HBOT-dedicated pumps	pre-HBOT (before HBOT)	Switching infusions to HBOT-dedicated pumps	0/19 (0%)	Infusions that must continue during HBOT are transferred to HBOT-compatible pumps according to local equipment practice.	Hyperbaric infusion-device literature discusses compatibility, fire-safety assessment, and performance of infusion pumps or syringe drivers under pressure; ICU-transfer checklist literature supports preparation and verification of infusion systems before movement [7,20,24].	HBOT-specific equipment constraint; local workflow requirement; retained despite zero respondent-reported observations.	Applicable only for infusions that continue during HBOT. If an infusion is intentionally paused, document and ensure post-HBOT restoration.
Checking for air bubbles in infusion lines	pre-HBOT (before HBOT)	Checking the presence of air bubbles in the infusion lines	3/19 (15.79%)	Infusion lines are checked before HBOT to remove visible air and confirm safe line preparation before exposure to pressure changes.	Air-embolism prevention literature supports avoiding intravascular air entry, and hyperbaric infusion-device literature supports line inspection after device preparation or pressure-related manipulation [3,7,24].	Survey signal; HBOT-specific pressure-related concern; local workflow requirement.	Applicable when IV lines are connected. The item does not quantify actual embolic risk; it is a visible-line workflow check.
Ensuring ECG, invasive blood pressure (IBP), and SpO_2 monitoring	pre-HBOT (before HBOT)	ECG, IBP, SpO_2 monitoring	1/19 (5.26%)	Monitoring continuity is confirmed before transfer and chamber preparation to maintain surveillance of a mechanically ventilated ICU patient.	ECHM/HBOT position statements and hyperbaric equipment reviews emphasise physiological monitoring for critically ill patients during HBOT; ICU-transfer guidance similarly identifies monitoring continuity as an essential transport requirement [2,7,20].	Core critical-care transfer requirement; local workflow requirement; survey signal present.	Applicable according to patient status and available HBOT-compatible monitoring. IBP applies when invasive arterial monitoring is present.
Disconnecting unnecessary or non-essential equipment	pre-HBOT (before HBOT)	Disconnecting unnecessary/unwanted equipment	2/19 (10.53%)	Non-essential devices are removed or disconnected to reduce clutter, incompatibility, transfer burden, and chamber-safety issues.	Hyperbaric guidance emphasises chamber-compatible materials and limitation of unnecessary equipment; ICU-transfer guidance and checklist-development literature support simplification to necessary devices and explicit equipment preparation before movement [1,7,21,23].	Survey signal; HBOT-specific equipment/environmental constraint; local workflow requirement.	Requires clinical judgement: essential life-support devices must not be disconnected. Document N/A if no non-essential equipment is present.
Disconnecting renal replacement therapy (RRT) (before HBOT when required)	pre-HBOT (before HBOT)	Disconnecting renal replacement therapy	0/19 (0%)	If renal replacement therapy is in progress, local workflow requires a deliberate decision to disconnect or manage it before HBOT according to patient status and equipment constraints.	Hyperbaric equipment literature identifies haemofiltration and other complex life-support technologies as challenging in the hyperbaric environment; ICU-transfer guidance supports explicit planning for therapies that cannot safely continue during transport or procedures [1,7,27].	Low-frequency, clinically relevant workflow item; local workflow requirement; retained despite zero respondent-reported observations.	Applicable only to patients receiving renal replacement therapy. Requires physician/nursing decision and local device-specific protocol.
Preparing the intubation set for transport	pre-HBOT (before HBOT)	Preparing the intubation set for transfer	14/19 (73.68%)	An intubation/rescue airway set is prepared before transfer because airway emergencies may require immediate action outside the usual ICU bedspace.	Critical-care transport guidance supports immediate availability of airway rescue equipment during movement of ventilated patients; hyperbaric critical-care literature highlights the need for dedicated support equipment and experienced staff near the chamber [4,7,21,27].	Highest-frequency respondent-reported vulnerability; airway rescue readiness; plausible clinical consequence if omitted.	Applicable to mechanically ventilated patients. Contents should match local emergency airway protocol and chamber-area availability.
Preparing the self-inflating manual resuscitator bag	pre-HBOT (before HBOT)	Preparing the self-inflating manual resuscitator bag	6/19 (31.58%)	A manual resuscitator is prepared before transfer to permit emergency ventilation if ventilator function, circuit integrity, or transfer logistics fail.	Critical-care transport guidance requires backup ventilation capability for ventilated patients; hyperbaric critical-care literature emphasises compatible respiratory equipment and recognises technical limitations of some hyperbaric ventilation configurations [7,21,27].	Frequent respondent-reported vulnerability; backup ventilation requirement; local workflow requirement.	Use HBOT-compatible/locally approved equipment where required. Check connection interfaces and oxygen source according to local protocol.

Checklist item	Phase	Corresponding survey item	Reported by respondents n/N (%)	Local workflow rationale	Literature/HBOT rationale	Reason retained	Applicability notes
Switching the patient to an HBOT-dedicated mechanical ventilator	pre-HBOT (before HBOT)	Switching the patient to a HBOT-dedicated mechanical ventilator	0/19 (0%)	Patients requiring mechanical ventilation are switched to an HBOT-compatible ventilator before chamber treatment according to local practice.	Hyperbaric critical-care literature addresses dedicated or compatible ventilators and technical limitations of ventilation in the chamber; mechanically ventilated HBOT cohorts support feasibility when equipment, staffing, and sedation are appropriate [2,6,7,28].	HBOT-specific technical requirement; local workflow requirement; retained despite zero respondent-reported observations.	Applicable to mechanically ventilated patients undergoing HBOT. Zero reports may reflect strong standardisation rather than absence of risk.
Connecting capnography monitoring	pre-HBOT (before HBOT)	Connecting capnography monitoring	3/19 (15.79%)	Capnography is connected to support ventilation monitoring during preparation, transfer, and/or HBOT according to local equipment availability.	Critical-care transport guidance supports ventilation monitoring during movement; hyperbaric equipment literature notes that end-tidal CO2 monitoring requires interpretation under pressure while physiological monitoring remains a central component of critical care [7,20,21,27].	Survey signal; ventilation-monitoring requirement; local workflow step.	Applicable when capnography is available and indicated. Terminology should be standardised in the manuscript as capnography or capnography.
Removal or replacement of hazardous materials	post-HBOT (after HBOT)	Replacing supportive items/materials previously removed from the patient's bed	5/19 (26.32%)	Items removed before HBOT are restored after return to the ICU configuration to re-establish routine care and patient support.	Checklist, handover, and critical-care transport literature supports structured post-procedure or post-transfer checks to maintain continuity of care and verify restoration after movement [11,21,23,29].	Post-HBOT restoration requirement; respondent-reported vulnerability; closes the pre-HBOT removal loop.	Applicable only for items intentionally removed or replaced before HBOT. Use checklist pairing to avoid omissions.
Disconnecting the electric pump from the anti-decubitus mattress	post-HBOT (after HBOT)	Reconnecting the anti-decubitus mattress pump	10/19 (52.63%)	After HBOT, the electric mattress pump must be reconnected to restore standard pressure-area care.	Post-transfer and handover literature supports explicit restoration of baseline support after procedures or transport; the HBOT pathway creates deliberate device interruptions that require structured reversal checks [11,21,23,29].	High-frequency respondent-reported vulnerability; restoration of standard ICU support; paired pre/post item.	Applicable when the pump was disconnected before HBOT. Important for longer ICU care trajectories but not a direct measure of pressure-injury outcomes.
Securing the gastric tube with a decompression bag	post-HBOT (after HBOT)	Reconnecting feeding to the gastric tube	0/19 (0%)	If enteral feeding was interrupted for HBOT, reconnection or deliberate continued withholding should be checked after the session.	Critical-care handover and checklist literature supports explicit review of interrupted therapies after transport or procedures; item applicability depends on whether feeding was running, paused, or clinically withheld [11,21,23,29].	Local restoration step; low-frequency workflow item relevant to care continuity; retained despite zero respondent-reported observations.	Applicable only when enteral feeding was running or planned before HBOT and is clinically appropriate after the session.
Suctioning secretions from the airway and oral cavity	post-HBOT (after HBOT)	Suctioning of secretions from the respiratory tract and oral cavity	1/19 (5.26%)	Post-session airway assessment includes suctioning when clinically indicated after transfer and treatment.	Critical-care transport guidance supports reassessment of airway status after movement; HBOT care of ventilated patients requires continued airway vigilance and appropriately trained staff [2,20,28].	Survey signal; local airway reassessment step; plausible relevance to post-session airway management.	Not mandatory for every patient. Mark N/A or not indicated when no secretion burden is present.
Checking fixation of the endotracheal tube	post-HBOT (after HBOT)	Checking fixation of the endotracheal tube	0/19 (0%)	Endotracheal tube position and fixation are rechecked after transfer/session to detect displacement or loosening related to movement or equipment changes.	Critical-care transport guidance supports reassessment of airway devices after movement; mechanically ventilated HBOT patients remain exposed to airway-device and equipment-transition issues requiring post-session verification [2,20,21,28].	Core airway-related workflow check; retained because omission could plausibly have clinically important consequences; retained despite zero respondent-reported observations.	Applicable to all intubated patients; adapt for tracheostomy tubes and local fixation methods.
Replacing air with fluid in the endotracheal tube cuff	post-HBOT (after HBOT)	Replacing fluid with air in the endotracheal tube cuff	11/19 (57.89%)	After HBOT, cuff medium is restored to standard ICU practice according to local protocol.	HBOT-specific cuff-medium exchange is a pressure-related adaptation; available HBOT cuff-pressure data support explicit post-session restoration and cuff-pressure verification rather than assumptions about cuff behaviour [2,25,27].	High-frequency respondent-reported vulnerability; HBOT-specific paired restoration item; plausible airway-management consequence if omitted.	Applicable when the cuff medium was changed before HBOT. Ensure cuff pressure/volume is checked according to local airway protocol.
Securing drainage systems with one-way valves	post-HBOT (after HBOT)	Securing drains/drainage systems with dedicated equipment	0/19 (0%)	Drainage systems are reassessed after HBOT to ensure secure configuration and restore routine ICU management.	Critical-care transport guidance supports reassessment of tubes and drains after movement; hyperbaric equipment literature adds device-compatibility and pressure-related considerations for connected systems [7,20].	Local restoration step; relevant device-related workflow item; retained despite zero respondent-reported observations.	Applicable only when drains are present. Device-specific requirements should be defined locally.
Switching infusions to HBOT-dedicated pumps	post-HBOT (after HBOT)	Switching intravenous infusions back to bedside syringe pumps	0/19 (0%)	Infusions transferred to HBOT-dedicated pumps are switched back to standard ICU pumps after HBOT to restore usual bedside delivery and alarms.	Hyperbaric infusion-device literature discusses device compatibility, pump performance, and change-over considerations; ICU-transfer and handover/checklist literature supports explicit restoration and verification of infusion devices after transport or procedures [7,23,24,29].	Paired pre/post workflow step; local restoration requirement; retained despite zero respondent-reported observations.	Applicable only when infusions were switched before HBOT. If infusions were paused, use the separate reconnection item.
Checking for air bubbles in infusion lines	post-HBOT (after HBOT)	Checking the presence of air bubbles in the infusion lines	3/19 (15.79%)	Infusion lines are rechecked after equipment transitions and pressure exposure before standard ICU care continues.	ICU-transfer guidance supports post-transfer verification of lines and infusion systems; air-embolism prevention literature and hyperbaric infusion-device considerations provide rationale for line inspection after pressure exposure and device transitions [20,21,30].	Survey signal; local infusion-line verification step; pressure-related concern.	Applicable when IV lines are connected. This is a workflow verification item, not a documented adverse-event measure.
Ensuring ECG, invasive blood pressure (IBP), and SpO_2 monitoring	post-HBOT (after HBOT)	ECG, IBP, SpO_2 monitoring	3/19 (15.79%)	Monitoring is re-established or confirmed after return from HBOT to restore standard ICU surveillance.	HBOT and ICU-transfer guidance emphasise continuous monitoring and reassessment of critically ill patients during and after transport or procedures; handover literature supports structured restoration checks to reduce monitoring gaps [2,20,27,29].	Survey signal; standard ICU restoration requirement; local workflow step.	Applicable according to patient monitoring needs and devices used. IBP applies when arterial monitoring is present.
Disconnecting unnecessary or non-essential equipment *and* Disconnecting renal replacement therapy (RRT)	post-HBOT (after HBOT)	Reconnecting previously disconnected equipment, including renal replacement therapy	0/19 (0%)	Equipment intentionally disconnected before HBOT must be deliberately reconnected or formally withheld after clinical reassessment.	Post-transfer and checklist literature supports deliberate restoration or documented withholding of baseline therapies after movement or procedures; hyperbaric equipment literature highlights limitations for complex devices such as haemofiltration [7,20,23,29].	Local restoration requirement; retained because omission could plausibly have clinically important consequences; retained despite zero respondent-reported observations.	Applicable only to equipment disconnected before HBOT. RRT reconnection requires clinical decision and local protocol.
Switching the patient to an HBOT-dedicated mechanical ventilator	post-HBOT (after HBOT)	Reconnecting the patient to the bedside mechanical ventilator	0/19 (0%)	After treatment, ventilatory support is returned to the usual ICU ventilator configuration according to local practice.	Hyperbaric critical-care literature addresses chamber-compatible ventilators and differences from standard ICU ventilation; ICU-transfer guidance supports confirmation of ventilation, settings, alarms, and patient tolerance after transfer [7,27,28].	HBOT-specific paired restoration item; local workflow requirement; retained despite zero respondent-reported observations.	Applicable to mechanically ventilated patients when an HBOT-dedicated ventilator was used. Confirm settings, alarms, circuit integrity, and patient tolerance.
Connecting capnography monitoring	post-HBOT (after HBOT)	Reconnecting capnography monitoring	5/19 (26.32%)	Capnography is reconnected after HBOT to restore standard ventilation monitoring in the ICU setting.	Critical-care transport and handover literature supports restoration of ventilation monitoring after movement; hyperbaric equipment literature notes specific interpretation issues for end-tidal CO2 during pressure exposure [7,20,27,29].	Respondent-reported vulnerability; monitoring restoration requirement; paired pre/post item.	Applicable when capnography is part of standard monitoring for the patient. Standardise terminology before submission.

Abbreviations: ECG, electrocardiography; HBOT, hyperbaric oxygen therapy; IBP, invasive blood pressure; ICU, intensive care unit; RRT, renal replacement therapy; SpO2, peripheral oxygen saturation.
